# Composition of the Essential Oil *Thymus schimperi* and Evaluation of Its Acute and Subacute Toxicity in Wistar Albino Rats: *In Silico* Toxicity Studies

**DOI:** 10.1155/2021/5521302

**Published:** 2021-07-23

**Authors:** Fentahun Adane, Kaleab Asres, Wondwossen Ergete, Samuel Woldekidan, Abiy Abebe, Boki Lengiso, Girma Seyoum

**Affiliations:** ^1^Department of Anatomy, College of Health Sciences, Addis Ababa University, Addis Ababa, Ethiopia; ^2^Department of Pharmaceutical Chemistry & Pharmacognosy, College of Health Sciences, Addis Ababa University, Addis Ababa, Ethiopia; ^3^Department of Pathology, School of Medicine, College of Health Sciences, Addis Ababa University, Addis Ababa, Ethiopia; ^4^Ethiopian Public Health Institute, Biomedical and Clinical Research Team, Traditional and Modern Medicine Research Directorate, P.O. Box 1242, Addis Ababa, Ethiopia; ^5^Directorate of Traditional and Modern Medicine Research, Ethiopian Public Health Institute, P.O. Box 1242, Addis Ababa, Ethiopia; ^6^Ethiopian Public Health Institute, National HIV/AIDS and TB Directorate, P.O. Box 1242/5654, Addis Ababa, Ethiopia

## Abstract

**Background:**

In Ethiopian traditional medicine, the aerial part of *Thymus schimperi* is widely used to treat diseases such as gonorrhea, cough, liver disease, kidney disease, hypertension, stomach pain, and fungal skin infections. However, there is insufficient investigation on the toxic effect of the essential oil of *T. schimperi*. The aim of this study was, therefore, to evaluate the acute, subacute, and *in silico* toxicity of *Thymus schimperi* essential oil in the Wistar albino rats.

**Method:**

Essential oil of the aerial part of *T. schimperi* extracted by hydrodistillation was analyzed by GC-MS. The oil was subjected to toxicity studies. In the acute toxicity study, rats were randomly divided into seven groups (*n* = 5). The control group received only distilled water with 2% of tween 80, whereas the experimental groups received single doses of 300, 600, 900, 1200, 1500, and 2 000 mg/kg of the oil. In the subacute toxicity study, rats were randomly divided into four groups (*n* = 10). The control group received distilled water with 2% of tween 80, whereas the experimental groups received 65 mg/kg, 130 mg/kg, and 260 mg/kg of the oil orally for 28 days. At the end of the experiment, blood samples were collected for hematology and clinical chemistry evaluation. Gross pathology and histopathology of the liver and the kidneys were also evaluated. For the *in silico* toxicity study, PubChem CID numbers of GC-MS identified bioactive compounds in the essential oil of *T. schimperi* obtained from PubChem. Chemdraw (8.0) was used to construct two-dimensional structures of the compounds. The Swiss ADMET web tool was used to convert the two-dimensional structures into a simplified molecular-input line input system (SMILES). In addition, the toxicity parameters were predicted via vNN and ADMET servers.

**Results:**

In this study, the LD_50_ of the essential oil of *T. schimperi* was found to be 1284.2 mg/kg. According to the World Health Organization, the oil is classified as moderately hazardous in its oral administration. In the subacute toxicity study, rats showed no significant changes in behavioral indices, gross pathology, body weight, biochemical, and most hematological parameters. However, hematological profiles showed a significant decrement in WBC counts and a significant increment of MCV in high dose (260 mg/kg) groups as compared to the control group. Furthermore, no significant differences were observed between the control and essential oil-treated groups, observed in the gross histopathology of the liver and the kidneys. In the *in silico* toxicity study, all compounds derived from the essential oil showed no cardiac toxicity (h-ERG Blocker), AMES (Ames Mutagenicity), and cytotoxicity via ADMET and vNN-ADMET toxicity predictors. However, by using these servers, about 8.6% of the compounds showed hepatotoxicity, only 3.45% caused drug-induced liver injury, and only 1.75% were potentially toxic to the mitochondrial membrane.

**Conclusion:**

From the results of this study, oral administration of the essential oil *T. schimperi* up to a dose of 130 mg/kg is not harmful. However, in the high-dose (260 mg/kg) group, the WBC count was significantly decreased and the MCV was significantly increased. In the *in silico* toxicity study, most of the components of the oil were found to be nontoxic, although a few of the compounds showed hepatotoxicity and mitochondrial membrane potential toxicity. It is, therefore, essential to conduct chronic toxicity of the essential oil as well as its components, which showed toxicity in the *in silico* study before using preparations containing the essential oil of *T. schimperi*.

## 1. Introduction

The genus *Thymus* contains about 350 species widely distributed in equatorial regions of the world [1]. In different parts of the world, *T. schimperi* (thyme) extracts are traditionally used orally to treat dyspepsia and other gastrointestinal disturbances, bronchitis, pertussis, laryngitis, tonsillitis, and coughs due to colds [2, 3]. Topical applications of thyme extracts have been used in the treatment of minor wounds, common cold, disorders of the oral cavity, and antibacterial agents in oral hygiene [3].

Members of the genus are one of the most widely used medicinal plants in Ethiopia, mainly because of their antimicrobial activity [2]. Both *T. schimperi* and *T.* s*errulatus* are locally known as “Tosign,” and their leaves are used as spices in various food products and medicines [4]. In Ethiopia, *T. schimperi* is used in a variety of forms [3]. The fresh or dried leaves are used locally as condiments and tea [5], in the preparation of “berbere”(pepper powder) and “shirro” (bean/pea powder) [6] and for the preparation of metata ayb (fermented cottage cheese) [7]. In traditional medicine, *T. schimperi* is used to treat different diseases like gonorrhea, cough, liver disease, renal diseases, hypertension [3], stomach pain [8], kidney problems [9], and dermal fungi [10].

*T. schimperi* contains about 1.0%–2.5% essential oil. The composition of the essential oil fluctuates depending on the chemo type of the plant. The principal components of *T. schimperi* are thymol and carvacrol (up to 64% of the oil), along with linalool, *p*-cymol, cymene, hymene, *α*-pinene, apigenin, luteolin, and 6-hydroxyluteolin glycosides, as well as di-, tri-, and tetra-methoxylated flavones [11].

Even though the leaves of *T. schimperi* are widely used in traditional medicine, there is not enough investigation on the toxic effects of the essential oil of the plant. Therefore, the purpose of this study was to evaluate the acute and subacute as well as *in silico* toxicities of the essential oil of *T. schimperi* in Wistar albino rats.

## 2. Materials and Methods

### 2.1. Plant Material

Fresh leaves of *T. schimperi* were collected in March 2019 from around Goba city, 400 km southeast of Addis Ababa, and 150 km east of Shashemene in Oromia Region. The plant material was authenticated by a botanist at the Ethiopian Public Health Institute (EPHI) where it was deposited (Collection number: HH-001) for future reference.

### 2.2. Study Animals

Wistar albino rats, 8 to 10 weeks of age, obtained from the Ethiopian Public Health Institute (EPHI) breeding unit, were utilized for this study. The female rats were nulliparous and nonpregnant. Same-sex rats were divided into experimental and control groups in a standard cage with five animals per group (*n* = 5) and held under standard conditions (at a temperature of 20°C (±2°C), with a normal 12-hour light/12-hour dark cycle). All the experiments were conducted following the internationally accepted laboratory animal use and care guidelines [12]. In addition, the Institutional Review Board (IRB) of the College of Health Sciences, Addis Ababa University, approved the protocol. Animals were acclimatized for one week before the commencement of the study and were provided with water and food pellets *ad libitum* before and until the end of the experimental period.

### 2.3. Essential Oil Distillation

Fresh leaves of *T. schimperi* (1 kg) were extracted by hydrodistillation using a Clevenger-type apparatus. The oil obtained was stored in a sealed amber-colored vial in a refrigerator at −10°C until it is used for the study.

### 2.4. Analysis of the Essential Oil

#### 2.4.1. GC Analysis

Separation was carried out on a Shimadzu gas chromatograph, model GC-14A, fitted with a supelcowax 10 (30 m × 0.25 mm, 0.2 *μ*m film thickness) fused silica column. The oven temperature was programmed as follows: 70°C (5 min), 70–180°C (5°C/min), 180–240°C (10°C/min), and 240°C (10 min). Helium was used as a carrier gas at a flow rate of 1 ml/min and with a split ratio of 82 : 1. Injector and Flame Ionization Detector (FID) temperatures were 210°C and 260°C, respectively.

#### 2.4.2. GC-MS Analysis

Qualitative GC-MS analyses were carried out using the Mass Lab VI.1 system equipped with an FI 8000GC. A Supelcowax 10 (30 m × 0.25 mm, 0.2 *μ*m film thickness) fused silica column was used with oven temperature programming: 60°C (5 min) and with the injector temperature at 210°C. Quantitative data were obtained by flame ionization detection and electronic integration without using FID response factors. The experiments were not replicated. The compounds were identified by coinjection (GC) with authentic samples and by computerized matching of the acquired mass spectra with library spectra (MS).

### 2.5. Acute Toxicity

Acute toxicity evaluation was performed in compliance with OECD 425 research guidelines [13]. Healthy female Wistar rats were fasted overnight but allowed access to water *ad libitum* and divided into seven groups (*n* = 5) randomly. Distilled water with 2% of tween 80 was provided to the first group (control group). The other six classes were treated orally with single doses of *T. schimperi* essential oil at 300, 600, 900, 1200, 1,500, and 2,000 mg/kg, respectively. Doses were selected after performing pilot studies. All the treatments were provided by force-feeding. Animals were examined for symptoms of toxicity and body weight, as well as mortality for 14 days. During the first 3 hours after essential oil administration, toxicity signs and symptoms were observed in individual cages and then evaluated regularly throughout the study [13]. The LD50 value was measured according to the rats' mortality observed within 14 days. On Day 15, all surviving animals were sacrificed, internal organs were excised, and organ weights were measured.

### 2.6. Subacute Toxicity

A subacute toxicity study was carried out in compliance with the recommendations of OECD 425 research guidelines [13]. The animals were randomly divided into four groups of 10 rats per group, each group containing five male and five female rats. The oil was administered by gavage orally in doses of 65 mg/kg, 130 mg/kg, and 260 mg/kg for 28 consecutive days, whereas only distilled water with 2% of tween 80 was given to the rats in the control group. The doses specified were based on the acute toxicity report LD50 value of 1284.2 mg/kg. Signs of toxicity and mortality were monitored regularly, with changes in body weight and weekly measurements of food and water intake. At the end of the study, animals were fasted overnight, diethyl ether anesthetized, and blood samples collected by cardiac puncture. Heparinized blood samples were used for the determination of hematological parameters. Nonheparinized tubes were used to analyze blood chemistry, while blood glucose was determined using fluoride tubes. The liver and the kidneys were removed and immediately weighed after dissection.

#### 2.6.1. Hematological and Biochemical Analyses

Ethylenediaminetetraacetic acid (EDTA) was used as a processor of blood samples in test tubes. Hematological parameters were determined on a hematology analyzer (SYSMEX XT-1800i, SYSMEX CORPORATION, Japan). White blood cell count (WBC), red blood cell count (RBC), hemoglobin concentration (HGB), hematocrit (HCT), mean corpuscular volume (MCV), mean corpuscular hemoglobin concentration (MCHC), and platelet count (PLC) were evaluated. For biochemical analysis, blood samples were allowed to stand for 3 hrs in plain test tubes for full clotting and centrifuged for 15 min at 5000 rpm using a benchtop centrifuge (Humax-k, Human-GmbH, Germany). The plasma was drained and transferred to other clean vials, and the serum was kept at −20°C until clinical biochemistry measurements were done. The concentrations of alanine aminotransferase (ALT), aspartate aminotransferase (AST), urea, albumin, and creatinine were automatically determined using Cobas Integra-400 plus Analyzer (Roche Diagnostics, Japan).

#### 2.6.2. Organ Weight Measurements and Tissue Samples

After assessing body weight, all experimental animals were sacrificed on day 29, and the target organs were taken. The organs were then kept for a few minutes in 1% normal saline to clean any extraneous tissues and weighed with precision balance. The tissue samples taken from the liver and the kidneys were placed in a test tube with 10% formalin buffered for 24 hrs and rinsed overnight under tap water. The fixed tissues were then dehydrated and washed with ethanol and xylene, respectively. In addition, it was infiltrated with molten paraffin wax and embedded in paraffin blocks. The blocks were sectioned at a thickness of 5–6 *μ*m using Leica rotary microtome (Leica RM 2125 RT, China, checked in Germany). Ribbons of the tissue sections were gently collected using forceps and placed on the surface of a water bath at 30–40°C before they were placed over the tissue. The slides were then mounted in slide racks and placed overnight in an oven at a temperature of 20–40°C to make it easy for the specimens to be fixed on the glass slides. The thin sections then underwent different stages of xylene and alcohol treatment and stained with hematoxylin and eosin [14].

#### 2.6.3. Light Microscopy and Photomicrography

Stained tissue portions of the liver and the kidney were carefully examined in a binocular compound light microscope (Olympus CX41, Japan). Sections of tissue from the treated groups were examined for any signs of histopathological changes. Photomicrographs of selected slides from both the treated and the control group were taken using an automated digital photo camera (Evos XI, China), under a magnification of ×40 and ×20, respectively.

#### 2.6.4. Data Processing and Analysis

All data presented with numbers were analyzed by SPSS statistical software. All values have been expressed in mean ± SEM (standard error of the mean). Treatments over time were compared by using a one-way analysis of variance (ANOVA) among control and treated groups followed by Dunnett's *t*-test to determine the significance level. Statistical significance was considered at *P* < 0.05.

### 2.7. Ethical Consideration

In this study, the Institutional Review Boards of the College of Health Sciences, AAU, and EPHI approved all procedures followed.

### 2.8. *In Silico* Toxicity Prediction

Compounds present in the essential oil of *T. schimperi* have been identified by GC-MS. The PubChem CID number was obtained from PubChem [15]. Chemdraw (8.0) [16] was used to build two-dimensional structures (Table 1). The Swiss ADME web tool was used to convert the two-dimensional structures into a simplified molecular-input line input system (SMILES) that can be analyzed by servers for toxicity prediction [17].

Toxicity profiles are as follows: hERG potassium channel inhibition (cardiotoxicity), H-HT (Human Hepatotoxicity), and AMES (Ames Mutagenicity) distribution were predicted using the ADMET (Absorption, Distribution, Metabolism, Execration, and Toxicity) server [18]. Drug-induced liver injury (DILI), mitochondrial membrane potential (MMP) toxicity, and cytotoxicity parameters were predicted via vNN server [19].

## 3. Results

### 3.1. Chemical Composition Analysis

**Figure 1:**
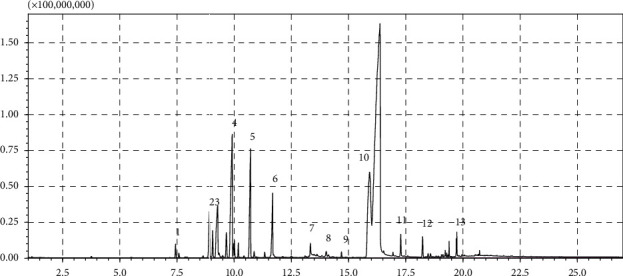
Gas chromatogram of the essential oil of *Thymus schimperi*.

Percentage yield of the essential oil of the fresh leaves of *T. schimperi* obtained by hydrodistillation was 1.39% (w/w). The oil was dark yellowish with a strong spicy scent. Qualitative and quantitative analyses carried out by GC/MS and GC identified 57 compounds representing 88.75% of the total essential oil. Results of the GC/MS analysis are summarized in Table 2, and GC chromatogram of the oil is depicted in Figure 1. As shown in Table 1, the major constituents of the oil were carvacrol (49.90%), thymol (10.64%), *o*-cymene (8.54%), *α*-terpinene (4.5%), linalool (2.51%), and 3-octanol (2.48%).

The result of acute toxicity of *T. schimperi* essential oil given orally in single doses is shown in Table 3. Starting at a dose of 900 mg/kg of the essential oil, the rats showed signs of toxicity, such as hypoactivity, piloerection, and convulsion including death. Mortality was observed in 900, 1200, 1500, and 2000 mg/kg groups with 20%, 20%, 80%, and 100% deaths, respectively (Table 3). The approximate LD50 obtained from the acute toxicity study was 1,284.2 mg/kg. There was a significant decrease in body weight in 900 mg/kg and 1200 mg/kg treated groups as compared to the control group at day 7 on the acute toxicity study (*P* < 0.05). In addition, on day 14, body weight has significantly decreased in 600 mg/kg, 900 mg/kg, and 1200 mg/kg treated groups (*P* < 0.05) as compared to the control group. Similarly, body weight has significantly decreased in the 1200 mg/kg treated group as compared to 300 mg/kg treated group (*P* < 0.05). Nonetheless, treatment groups (1500 mg/kg and 2000 mg/kg) were not included in the analysis as these groups had too few and no living rats, respectively. Furthermore, the weights of the kidney and the liver significantly increased in treatment groups (900 mg/kg and 1200 mg/kg) as compared to the control group (Table 4).

### 3.2. Subacute Toxicity Study

In the subacute toxicity study, rats were randomly assigned to four groups, each of the groups containing 10 rats (5M and 5F). Rats in the control group received distilled water with 2% of tween 80, while the experimental groups received 65 mg/kg, 130 mg/kg, and 260 mg/kg of essential oil orally for 28 days. Neither signs of toxicity nor deaths were observed after *T. schimperi* essential oil administration. *T. schimperi* essential oil did not result in any major changes in the body and organ weights (Table 5).

#### 3.2.1. Hematological and Biochemical Parameters

Hematological evaluation has shown a significant decrement in WBC counts and increment in the MCV in the high dose group (260 mg/kg) as compared to the control group. There was no significant difference in RBC, HB, HCT, MCH, MCHC, and PLT levels between the control group and any of the experimental groups (Table 6).

There were no significant differences in liver injury markers (ALT, AST, and ALP) between the control and any of the treatment groups. In addition, there were no significant changes in levels of blood urea and creatinine, which are indicators of kidney injury. Similarly, there was no significant difference in the levels of HDL and LDL between the control and treatment groups. Finally, electrolytes analysis revealed no significant differences in blood electrolyte levels such as sodium and potassium levels between the control and the treatment groups (Table 7).

#### 3.2.2. Morphological Analysis

**Figure 2:**
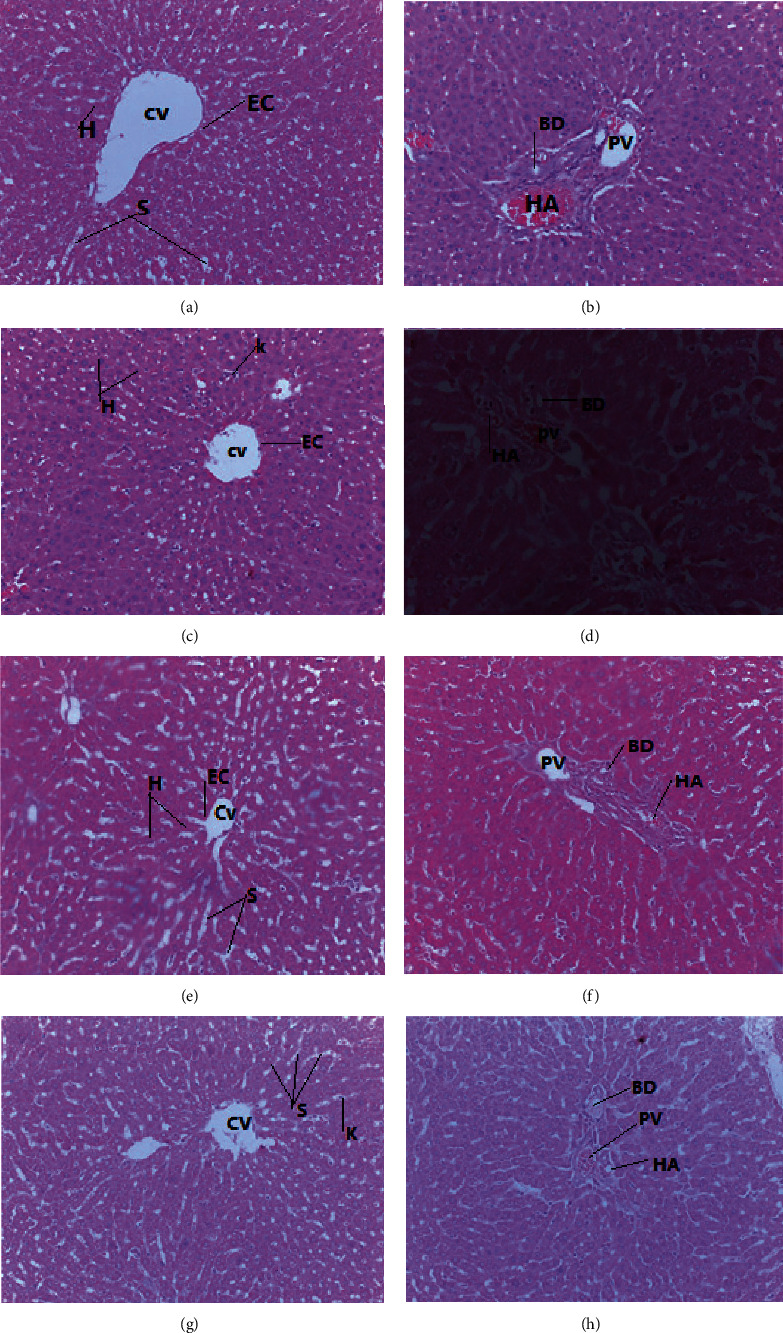
(a, b) Photomicrographs of liver sections of control rats; (c, d) liver sections of rats treated with 65 mg/kg of essential oil of *Thymus schimperi*; (e, f) liver sections of rats treated with 130 mg/kg of essential oil of *Thymus schimperi*; and (g, h) liver sections of rats treated with 260 mg/kg of essential oil of *Thymus schimperi*.

**Figure 3:**
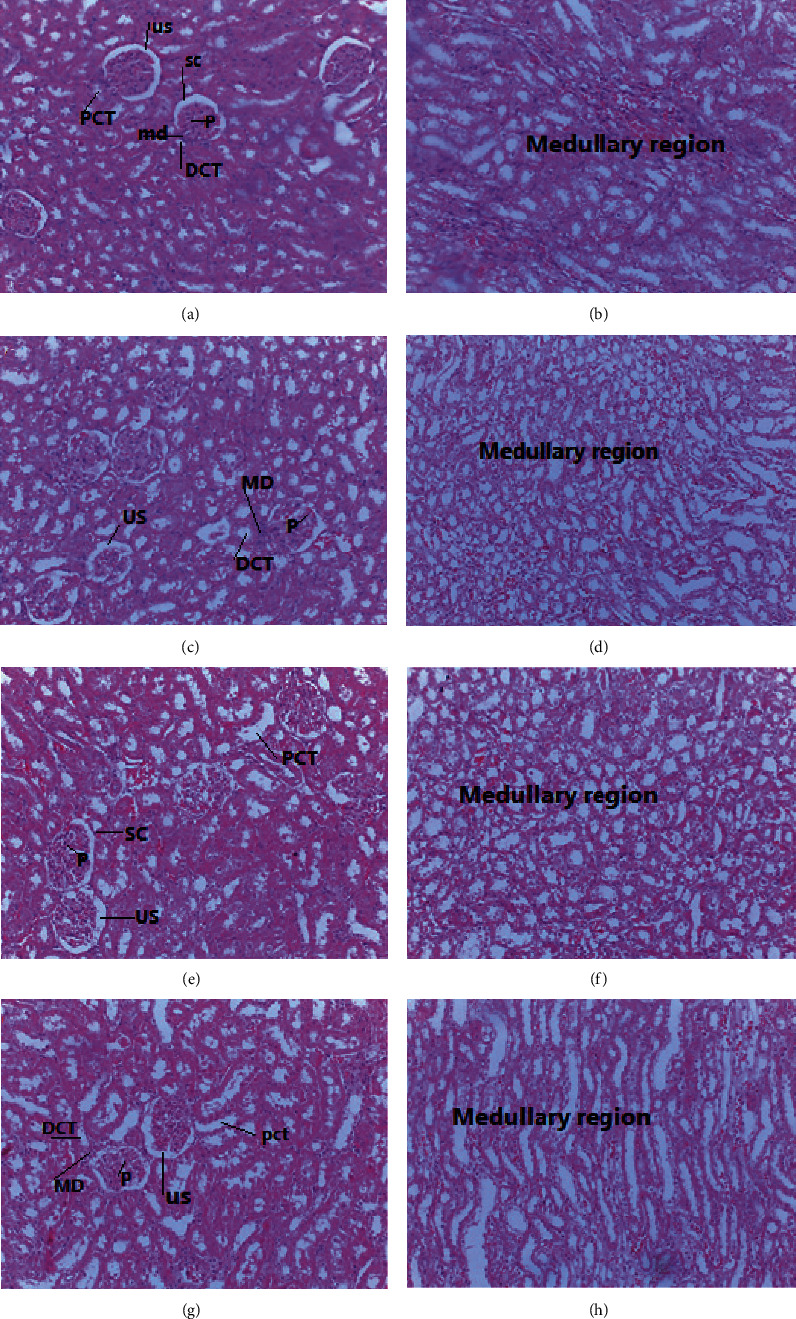
(a, b) Photomicrographs of the kidney sections of control rats, (c, d) kidney sections rats treated with 65 mg/kg of essential oil of *Thymus schimperi*, (e, f) kidney sections of rats treated with 130 mg/kg of essential oil of *Thymus schimperi,* and (g, h) kidney sections of rats treated with 260 mg/kg of essential oil of *Thymus schimperi*.

In the selected organs, the gross pathological analysis showed no observable irregularities. Furthermore, the histopathological analysis detected noticeable abnormalities in neither the control nor the treatment groups (Figures 2 and 3).

### 3.3. *In Silico* Toxicity Prediction of Compounds from the Essential Oil of *T. schimperi*

Toxicities of compounds from the essential oil were also tested by ADMET and vNN-ADMET servers. Toxicity and toxicological endpoint findings showed that all compounds derived from *T. schimperi* essential oil were free of h-ERG Blocker (cardiac toxicity), AMES (Ames Mutagenicity), and cytotoxicity. Regarding the hepatotoxicity parameters, most of the compounds (91.4%) did not show any toxicity, while 8.6% of the compounds showed hepatotoxicity. Compounds that have hepatotoxicity effects are *trans*-sabinol, methyl m-tolyl carbinol, 2-isobutylidene amino-3-methyl butyronitrile, limonene-10-ol, and *ß*-atlantol. Most of the compounds (96.55%) were safe for DILI (drug-induced liver injury). However, 3.45% of the compounds (thymol acetate and carvacrol acetate) have shown DILI toxicity. In addition, most of the compounds have not shown mitochondrial membrane potential (MMP) toxicity, except thymol (Table 8).

## 4. Discussion

Various medicines of herbal origin have widely been used around the world as primary therapies for various diseases [20]. Safety is checked by conducting general preclinical toxicity experiments to detect potential toxic effects of any drug, primarily in the liver and kidneys of animals [21]. If these organs are found to be mildly inflamed and damaged, the cell membrane permeability will significantly increase releasing cytoplasmic enzymes such as ALP and AST in the blood. Similarly, inflammation results in the release of mitochondrial ALT and AST [22, 23]. Models for the toxicity screening give valuable preliminary data which can help identify natural remedies with possible health benefits [24].

Major constituents of the essential oil of *T. schimperi* leave were carvacrol, thymol, *O*-cymene, and *α*-terpinene. The chemical composition of the oil was similar to the one previously reported by Asfaw et al. [25], which identified p-cymene, *γ*-terpinene, thymol, and carvacrol as major components of the oil.

In this study, using probit analysis, the LD_50_ of *T. schimperi* essential oil was found to be 1284.2 mg/kg. This level of LD_50_ is considered moderately hazardous, in oral use, as per WHO suggestions of pesticides guidelines [26]. This finding was slightly lower than the report from a study conducted in Debre Berhan, Ethiopia (LD_50_ value of 2000 mg/kg) [27]. The possible justification for this slight discrepancy could be due to the animal model difference that in this study Wistar albino rats were used, while mice were used in the previous study. The present acute toxicity study also revealed that *T. schimperi* essential oil induced hypoactivity, piloerection, convulsion, and irregular body movements in the tested animals. This finding is consistent with a study done by Dires et al. [27], which reported that administration of a single oral dose of the essential oil of *T. schimperi* causes signs of toxicity, such as hypoactivity, piloerection, and convulsion that may have resulted from disruptions in the activity of the autonomic nervous system (ANS) and the central nervous system (CNS). In an acute toxicity study, *T. schimperi* essential oil induced a substantial drop in body weight at higher doses which may be linked with the adverse symptoms causing the rats to become anorectic [28]. In the current study, the increment in the weight of the kidneys and the liver is most likely due to edema [29].

The hematological system is susceptible to toxic chemicals and can be used as a significant index for detecting human and animal physiological changes [30]. Hematological tests can quickly show physiological changes in the body, and the blood sample usually provides valuable information on the body's reaction to injury or disease, hunger, and stress [31]. The extent of the toxic effect of drugs and/or plant extracts can therefore be determined by evaluation of hematological parameters [32].

In the current study, there was a significant decrease in the mean white blood cell (WBC) count at a dose of 260 mg/kg as compared to the rats in the control group. This could be due to the effects of the major bioactive compounds in *T. schimperi* essential oil, like carvacrol and thymol, and which could cause cell cycle to arrest in the sub-G0/G1 phase, cellular apoptosis, and cell proliferation [33, 34]. There was also an increment in the mean corpuscular volume (MCV), the index that helps to determine the size of erythrocytes, at a dose of 260 mg/kg. This could be because any substance that affects cellular DNA biosynthesis, either directly or indirectly, can cause macrocytic changes. MCV elevation is a sign of alterations in DNA biosynthesis [35]. A previous study indicated that carvacrol inhibits DNA synthesis [36].

In the toxicological assessment, biochemical parameters play a significant role as markers due to their response to clinical signs and symptoms caused by toxicants. Assessment of liver and kidney function has paramount importance to determine the toxic properties of extracts and drugs [37]. In the present study, treatment of the animals with *T. schimperi* essential oil did not result in a significant change of all biochemical parameters. Any damage to the liver causes both ALT and AST to rise in the blood and could be taken as the first sign of the damage [37]. Creatinine level is known as a strong measure of renal function. An increase in creatinine means that there is noticeable harm to functioning nephrons [37, 38]. AST is primarily found in red blood cells, cardiac and skeletal muscles, and the kidneys. AST is not as specific to the liver as ALT. In the present study, the mean values of ALT and ALP in treatment groups increased, while AST decreased across treatment groups compared to control; however, the changes were not statistically significant. This result was found to be consistent with a reported data from a similar study previously conducted on the same plant from Ethiopia [27]. In addition, the constituents of the essential of *T. schimperi* did not show any cardiac toxicity (h-ERG Blocker), AMES (Ames Mutagenicity), and cytotoxicity by ADMET and vNN-ADMET toxicity prediction servers. Another justification for these results might be that there are very few compounds that can cause hepatotoxicity or drug-induced liver injury, as revealed by *in silico* toxicity studies (8.6% of the total compounds were hepatotoxic, and only 3.45% were caused by drug-induced liver injury and only thymol had potential toxicity to the mitochondrial membrane toxicity).

Plasma urea measurement has been used for many years as a marker of renal function. Plasma urea is usually increased in acute and chronic kidney disease. Urea removal falls as the kidney fails and, as a result, urea tends to accumulate with diseased kidneys that are unable to excrete these substances at normal rates; this will increase the level of urea in the blood [37, 39]. The average adult rat serum urea was measured approximately 15–45 mg/dl [40]. In the present study, mean urea values were shown to be slightly increased at doses of 65 mg/kg and 260 mg/kg, although not significant, and were not associated with histopathological changes in the kidneys.

Creatinine is formed in an endogenous manner and released at a constant rate into body fluids, and its plasma concentration is mainly controlled by glomerular filtration. As a result, both plasma concentration and its renal clearance were used as measures of the glomerular filtration rate [41]. The mean amount of creatinine in the current study showed a slight increase but was not significant. In adult rats, the reference value for creatinine is around 0.2–0.8 mg/dL [42]. The measurement in this analysis was within the reference value and was supported by a lack of histopathological changes in the kidneys.

The increment of total serum protein is caused by a change in plasma water volume and an increase in plasma concentrations of one or more different proteins. Decreased plasma water volume is observed in cases of dehydration due to inadequate water intake or excessive water loss, such as severe vomiting or diarrhea [41]. The standard value of total protein in adult rat serum is 5.6–7.6 mg/dL [40]. Throughout the treatment groups, the overall protein levels were slightly higher when compared to the control, but it was not statistically significant. The mean total protein values for rats were within the normal range.

Lipid profile is the term given for the evaluation of total cholesterol, triglycerides, lipoproteins of high density (HDL), and lipoproteins of low density (LDL). This test is commonly used to diagnose hyperlipidemia, a risk factor for heart disease [43]. However, the results of this study did not show a significant change in any of the components listed above. This finding was also supported by the result that all compounds extracted from *T. schimperi* essential oil were found to be free of h-ERG Blocker (cardiac toxicity) through ADMET and vNN-ADMET toxicity prediction servers.

The electrolytes found in blood and other body fluids are sodium and potassium. They help maintain the body's water and electrolyte balance and are also important for the proper functioning of the nerves and muscles. The hormone aldosterone controls the levels of sodium and potassium in the body. These electrolytes do not have significant changes based on the findings of this study. They are also within the normal range, in both treated and control groups.

Histopathological evaluations provide information on biochemical and hematological parameters to be improved [44]. Compared to controls, the general architecture of the liver, the appearance of the hepatocytes, the hepatic sinusoids, the portal triads, and the central veins are normal. Furthermore, compared to the control, the general histological architecture was not compromised in any of the treatment groups. The no-significant change of histopathological parameters of the liver between the control animals and the test animals after 4 weeks of treatment indicates that the essential oil did not cause adverse toxic effects or hepatic damage to the liver, and this result is consistent with other studies [27, 45].

In kidney histopathology analysis, rats treated with the essential oil showed no significant difference compared to controls. The sections of the treated rat kidneys displayed normal general renal structure and the regular presence of glomeruli and tubules. The proximal tubules, the distal tubules, and the macula densa were normal. The finding was further confirmed by the values of the blood's biochemical parameters (such as urea, creatinine, and total protein), which are the principal markers of kidney damage [46]. This was consistent with the previous study, which stated that there was no difference in tissue morphology between the control group and treatment groups [27].

In addition to the in vivo toxicity study on animal models, the toxicity profile of all the compounds of *T. schimperi* essential oil was also evaluated by ADMET and vNN-ADMET servers [19, 47]. Constituents of *T. schimperi* essential oil safety and toxicological findings showed that h-ERG Blocker (cardiac toxicity), AMES (Ames Mutagenicity), and cytotoxicity are free of toxicity. Regarding the hepatotoxicity parameter, most of the compounds (91.4%) did not show toxicity, although hepatotoxicity was seen in 8.6% of the compounds (*trans*-sabinol, methyl *m*-tolyl carbinol, 2-isobutylideneamino-3-methyl butyronitrile, limonene-10-ol, and *ß*-atlantol). Furthermore, most of the compounds (96.55%) were safe for DILI (drug-induced liver injury); however, 3.45% (thymol acetate and carvacrol acetate) have shown DILI toxicity. Finally, most of the compounds have not shown mitochondrial membrane potential (MMP) toxicity, except thymol.

## 5. Conclusion and Recommendations

The yield of the essential oil from the aerial part of *T. schimperi* was found to be 1.39% v/w. GC-MS study of the oil enables the identification of 57 compounds. Carvacrol was the major component of the essential oil, representing 49.90% followed by thymol (10.64%). Acute toxicity study showed that the LD_50_ of the oil was 1284.2 mg/kg. Similarly, subacute toxicity study demonstrated that the oil of *T. schimperi* does not adversely affect body weight, biochemical, and most hematological parameters at the tested doses, although the WBC count was significantly decreased and the MCV was significantly increased at a dose of 260 mg/kg. Besides, there were no signs of toxicity shown in the kidney and liver sections of the treated rats. All constituents of the essential oil of *T. schimperi* did not show any cardiac toxicity (h-ERG Blocker), AMES (Ames Mutagenicity), and cytotoxicity by ADMET and vNN-ADMET toxicity predictors. However, 8.6% of the compounds were hepatotoxic, and only 3.45% were caused by drug-induced liver injury, and only 1.75% has potential toxicity to the mitochondrial membrane. Based on this study, oral administration of the essential oil *T. schimperi* up to a dose of 130 mg/kg is not harmful. However, in the high-dose (260 mg/kg) group, the WBC count was significantly decreased and the MCV was significantly increased. In the *in silico* toxicity study, most of the components of the oil were found to be nontoxic, although few of the compounds showed hepatotoxicity and mitochondrial membrane potential toxicity. It is therefore essential to conduct chronic toxicity study on the essential oil as well as its components, which showed toxicity in the *in silico* study before using preparations containing *T. schimperi* essential oil as drugs.

## Figures and Tables

**Table 1:** Structure of compounds from *Thymus schimperi* essential oil.

Compounds	Chemical structure	Compounds	Chemical structure
Butanoic acid, 2-methyl-, methyl ester	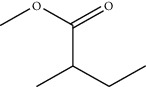	*γ*-Amorphene	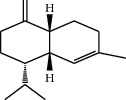
*α*-Thujene	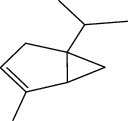	Neryl isobutanoate	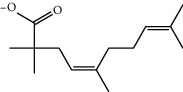
*α*-Pinene	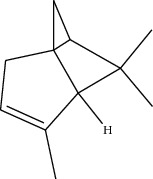	Viridiflorene	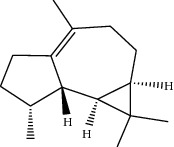
1,3,5-Cycloheptatriene, 7-ethyl-	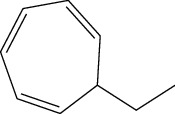	Geranyl isobutanoate	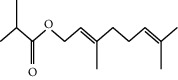
Camphene	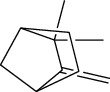	*β*-Sesquiphellandrene	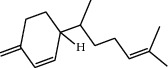
*β*-Pinene	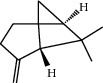	*β*-Vetivenene	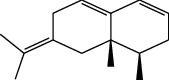
2,3-Diazabicyclo [2.2.2] oct-2-ene	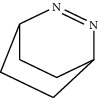	*β*-Atlantol	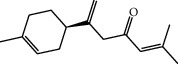
3-Octanone	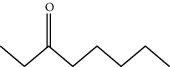	Methyl 11,12-octadecadienoate	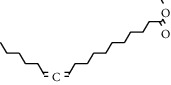
Myrcene	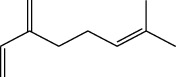	Tetracosane	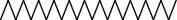
3-Octanol	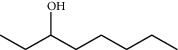	Carvacrol acetate	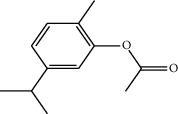
Phellandrene<alpha->	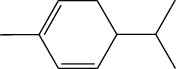	Sesquithujene<7-epi->	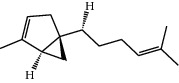
Carene<delta-3->	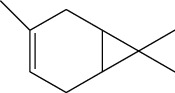	*β*-Bourbonene	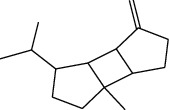
Cymene<ortho->	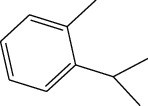	Caryophyllene (*E*-)	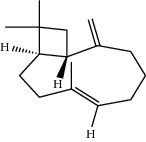
D-limonene	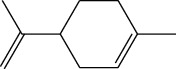	*β*-Gurjunene	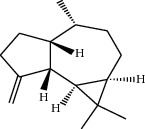
Cineole<1, 8->	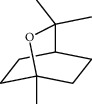	*trans*-Alpha-bergamotene	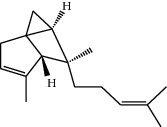
*β*-Z-ocimene	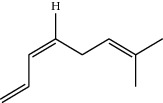	Aromadendrene<allo->	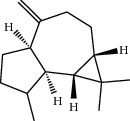
*α*-Terpinenes	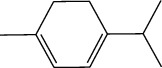	3,5-Methanocyclopentapyrazole, 3,3a,4,5,6,6a-hexahydro-3a,4,4-trimethyl-	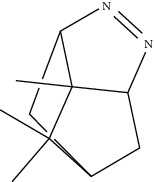
*γ*-Terpinene	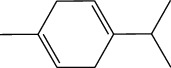	Carvacrol, methyl ether	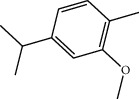
Dihydromyrcenol	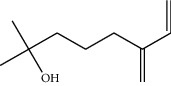	Neral	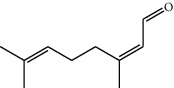
Carvacrol	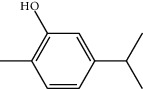	Dodecane	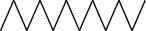
Thymol acetate	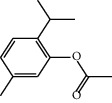	Dihydrocarvone<*trans*->	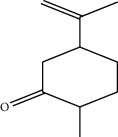
3,5-Methanocyclopentapyrazole, 3,3a,4,5,6,6a-hexahydro-3a,4,4-trimethyl-	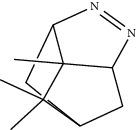	Terpinolene	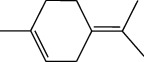
1,4-Methano-1H-cyclopenta[d]pyridazine, 4,4a,5,7a-tetrahydro-8,8-dimethyl-, (1. alpha,4alpha,4a. alpha,7a. alpha)-	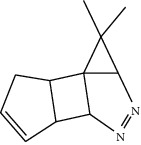	Linalool	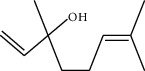
Sabinol<*trans*-> (*trans* for OH versus IPP)	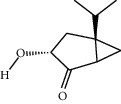	Sabina ketone<dehydro->	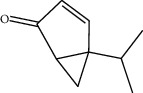
Isoborneol	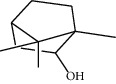	*α*-1-Campholena	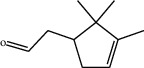
Methyl m-tolyl carbinol	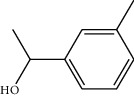	Ocimene<allo->	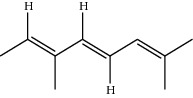
Terpineol<alpha->	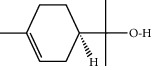	Limonen-10-ol	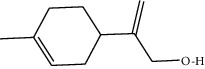
Cyclohexane, 1-butenylidene-	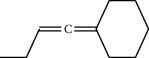	Thymol	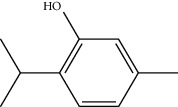
2-Isobutylideneamino-3-methylbutyronitrile	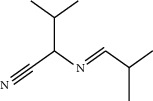		

**Table 2:** Composition of the essential oil of the fresh leaves of *Thymus schimperi*.

No.	Compounds	Percent	Ret. time	Ret. index
1	Butanoic acid, 2-methyl-, methyl ester	0.05	3,787	712
	*α*-Thujene	0.34	7,436	921
	*α*-Pinene	0.12	7,585	925
	1,3,5-Cycloheptatriene, 7-ethyl-	0.03	7,861	936
	Camphene	0.03	7,939	939
	*β*-Pinene	0.05	8,640	974
	2,3-Diazabicyclo[2.2.2] oct-2-ene	0.02	8,818	977

2	3-Octanone	1.04	8,889	980
	Myrcene	0.58	9,048	987

3	3-Octanol	2.48	9,213	993
	*α*-Phellandrene	0.24	9,334	999
	*δ*-3-Carene	0.80	9,638	1012

4	*o*-Cymene	8.54	9,838	1020
	D-Limonene	0.30	9,935	1024
	1-8-Cineole	0.39	9,973	1026
	*β*-*Z*-Ocimene	0.39	10,166	1034

5	*α*-Terpinene	4.53	10,661	1055
	*γ*-Terpinene	0.28	10,860	1064
	Dihydromyrcenol	0.07	10,972	1069
	Terpinolene	0.20	11,331	1084

6	Linalool	2.51	11,633	1097
	Dehydrosabina ketone	0.03	12,099	1117
	*α*-Campholenal	0.03	12,132	1119
	Ocimene<allo->	0.04	12,253	1123
	Sabinol<*trans*-> (*trans* for OH vs. IPP)	0.11	12,491	1135
	Isoborneol	0.12	13,102	1165

7	Methyl *m*-tolylcarbinol	0.84	13,323	1176
	*α*-Terpineol	0.22	13,615	1190
	Cyclohexane, 1-butenylidene-	0.35	13,727	1195
	2-Isobutylideneamino-3-methylbutyronitrile	0.09	13,787	1198
	Dodecane	0.10	13,843	1201
	*trans-*Dihydrocarvone	0.09	13,945	1206
	3,5-Methanocyclopentapyrazole, 3,3a,4,5,6,6a-hexahydro-3a,4,4-trimethyl-	0.07	14,124	1215
	1,4-Methano-1H-cyclopenta[d]pyridazine, 4,4a,5,7a-tetrahydro-8,8-dimethyl-, (1. alpha.,4. alpha.,4a. alpha.,7a. alpha.)-	0.03	14,350	1226
	Carvacrol, methyl ether	0.15	14,687	1242
	Neral	0.03	14,759	1248
	Limonen-10-ol	0.04	15,400	1277

8	Thymol	10.64	15,841	1298
	Carvacrol	49.90	16,130	1313
	Thymol acetate	0.08	16,859	1350

9	Carvacrol acetate	0.41	17,205	1369
	7-*epi*-Sesquithujene	0.04	17,384	1379
	*β*-Bourbonene	0.03	17,552	1387

10	*E-*Caryophyllene	0.53	18,201	1422
	*β*-Gurjunene	0.02	18,358	1435
	*trans*-*α*-Bergamotene	0.09	18,454	1443
	Aromadendrene<allo->	0.08	18,560	1452
	3,5-Methanocyclopentapyrazole, 3,3a,4,5,6,6a-hexahydro-3a,4,4-trimethyl-	0.03	18,809	1473
	D-Germacrene	0.03	18,907	1482
	*γ*-Amorphene	0.05	19,051	1494
	Neryl isobutanoate	0.07	19,118	1500
	Viridiflorene	0.15	19,218	1508
	Geranyl isobutanoate	0.06	19,288	1515
	*β*-Sesquiphellandrene	0.20	19,389	1523

11	*β*-Vetivenene	0.49	19,700	1560
	*β*-Atlantol	0.13	19,951	1614
	Methyl 11,12-octadecadienoate	0.30	20,535	1819
	Tetracosane	0.09	22,097	2402
Total (identified)		88.75%		

**Table 3:** Acute toxicity of single oral doses of *Thymus schimperi* essential oil in rats.

Conc. (C) (mg/kg)	Log (C)	Alive (%)	Dead (%)	Prop., p	Corr., p	Logit (*p*)	Probit (*p*)	Symptoms
0		100	0	0				None
300	2.5	100	0	0				None
600	2.8	100	0	0				None
900	3.0	80	20	0.2	0.2	−1.4	3.6	Hypoactivity, piloerection, convulsion
1200	3.1	80	20	0.2	0.2	−1.4	3.6	Hypoactivity, piloerection, convulsion
1500	3.2	20	80	0.8	0.8	1.4	6.4	Hypoactivity, piloerection, convulsion
2000	3.3	0	100	1				Hypoactivity, piloerection, convulsion
					Slope	11.9	11.9	
					Intercept	−37.0	−32.0	
					Test value	0.0	5.0	
					Log (C%)	3.1	3.1	
					LD 50	1284.2	1284.2	

After the dose, all rats treated were carefully examined for signs of toxicity and lethality up to 14°d.Conc. (C): concentration, log (C): logarithm of the concentration, alive (%): number of live rats in percent, and dead: number of dead rats in percent.

**Table 4:** Effects of different oral single doses of *Thymus schimperi* essential oil in rats for acute toxicity.

Parameters	Control	*T. schimperi* treatment of essential oil			
		300 mg/kg	600 mg/kg	900 mg/kg	1200 mg/kg
*Bodyweight loss (%)*					
Day 7	5.78 ± 0.07	5.00 ± 0.05	4.71 ± 0.03	3.43 ± 0.03^a^	2.24 ± 0.05^a^
Day 14	10.02 ± 0.11	7.01 ± 0.04	6.30 ± 0.04^a^	5.23 ± 0.04^a^	3.85 ± 0.05^b^

*Organs' weight (g)*					
Liver	6.72 ± 1.01	6.71 ± 1.08	7.45 ± 1.06	7.87 ± 0.08^b^	7.91 ± 0.09^b^
Kidney	1.52 ± 0.04	1.47 ± 0.03	1.58 ± 0.05	1.81 ± 0.06^b^	1.94 ± 0.06^b^
Heart	0.43 ± 0.05	0.42 ± 0.04	0.40 ± 0.03	0.41 ± 0.04	0.41 ± 0.04
Spleen	0.52 ± 0.09	0.55 ± 0.08	0.54 ± 0.07	0.55 ± 0.08	0.62 ± 0.08^a^

Data are expressed as mean ± SEM, *n* = 5 for each group; ^a^significant at *P* < 0.05 compared to the control only; ^b^significant at *P* < 0.05 compared to the control and 300 mg/kg group; ^c^significant at *P* < 0.05 compared to the control, 600, and 9 000 mg/kg; ^d^significant at *P* < 0.05 compared to the 900 and 1200 mg/kg.

**Table 5:** Body and organ weights of rats in the control and *T. schimperi* essential oil-treated groups in the subacute toxicity study.

Parameters	Control	Treatment of *T. schimperi* essential oil		
		65 mg/kg	130 mg/kg	260 mg/kg
Day 0	201.00 ± 2.78	198.00 ± 2.22	199.00 ± 1.98	198.00 ± 2.02
Day 7	207.00 ± 4.23	206.00 ± 2.50	207.00 ± 2.90	202.50 ± 2.42
Day 14	210.50 ± 4.41	210.00 ± 2.61	210.50 ± 3.56	205.50 ± 2.81
Day 21	212.50 ± 4.86	211.00 ± 3.67	212.00 ± 3.62	211.50 ± 4.56
Day 28	215.50 ± 4.89	215.00 ± 3.45	213.00 ± 4.71	213.50 ± 3.65

Organs' weight (g)				
Liver	5.58 ± 0.68	6.91 ± 0.20	6.47 ± 0.49	5.48 ± 0.65
Kidney	1.47 ± 0.15	1.20 ± 0.10	1.56 ± 0.06	1.40 ± 0.12
Heart	0.85 ± 0.06	0.68 ± 0.09	0.88 ± 0.02	0.72 ± 0.04
Spleen	0.56 ± 0.08	0.47 ± 0.09	0.46 ± 0.10	0.64 ± 0.07
Pancreas	1.14 ± 0.31	1.00 ± 0.20	1.01 ± 0.15	1.00 ± 0.33

The dataareexpressed as mean ± SEM,*n* = 10 for each group. There was no statistical differencebetweenthe control and the*T.schimperi*essential oil treatment groups (*P* > 0.05).

**Table 6:** Hematological values of rats in the control and *T. schimperi* essential oil-treated groups in the subacute toxicity study.

Parameter	Control	Treatment of *T. schimperi* essential oil		
		65 mg/kg	130 mg/kg	260 mg/kg
RBC (×10^6^/*μ*L)	8.50 ± 0.15	7.96 ± 0.20	8.04 ± 0.19	7.25 ± 1.22
WBC (×10^3^/*μ*L)	14.09 ± 1.37	12.94 ± 1.04	11.80 ± 1.50	7.40 ± 1.79^a^
Hb (g/dl)	16.70 ± 0.31	15.82 ± 0.30	16.28 ± 0.39	14.48 ± 2.29
HCT (%)	47.73 ± 0.84	47.98 ± 0.69	48.37 ± 1.36	41.97 ± 7.03
MCV (pg)	56.13 ± 0.49	57.57 ± 1.70	58.93 ± 0.80	60.15 ± 0.39^a^
MCH (pg)	19.63 ± 0.16	19.87 ± 0.17	20.25 ± 0.19	20.80 ± 0.97
MCHC (g/dl)	34.98 ± 0.54	32.97 ± 0.67	33.70 ± 0.33	35.68 ± 1.46
PLT ((×10^3^/*μ*L)	942.00 ± 120.50	952.00 ± 162.87	830.00 ± 79.07	804.50 ± 156.68

The data are expressed as mean ± SEM, *n* = 10 for each group. ^a^Significant difference compared to the control group (*P* < 0.05). RBC: red blood cells; WBC: white blood cells; Hb: hemoglobin; HCT: hematocrit; MCV: mean corpuscular volume; MCH: mean corpuscular hemoglobin; MCHC: mean corpuscular hemoglobin concentration; PLT: platelets.

**Table 7:** Blood chemistry values of rats in the control and *Thymus schimperi* essential oil-treated groups in the subacute toxicity study.

Parameter	Control	Treatment of *T. schimperi* essential oil		
		65 mg/kg	130 mg/kg	260 mg/kg
Urea (Mg/dL)	37.10 ± 2.37	39.97 ± 1.37	37.25 ± 0.89	40.13 ± 1.73
Creatinine (Mg/dL)	0.30 ± 0.01	0.32 ± 0.01	0.32 ± 0.01	0.35 ± 0.02
Sodium (mEq/L)	146.17 ± 0.54	147.33 ± 0.56	147.83 ± 1.28	146.17 ± 0.60
Potassium (mEq/LL)	4.43 ± 0.24	3.95 ± 0.20	4.55 ± 0.28	4.43 ± 0.46
Calcium (mEq/L)	2.33 ± 0.15	2.42 ± 0.31	2.41 ± 0.58	2.35 ± 0.30
Chloride (mEq/L)	105.17 ± 0.54	104.17 ± 0.54	105.17 ± 0.98	104.33 ± 0.33
Phosphate (mEq/L)	2.44 ± 0.62	2.26 ± 0.19	2.42 ± 0.13	2.38 ± 0.15
ALT (U/L)	52.38 ± 4.56	54.07 ± 5.78	65.60 ± 3.55	56.67 ± 2.15
AST (U/L)	216.35 ± 28.53	212.73 ± 16.28	200.13 ± 10.36	183.97 ± 13.40
ALP (U/L)	76.00 ± 10.35	73.33 ± 11.49	91.00 ± 7.23	88.00 ± 7.33
Albumin (g/dL)	4.26 ± 0.09	4.38 ± 0.11	4.44 ± 0.15	4.12 ± 0.17
Total protein (g/dL)	5.76 ± 0.09	5.85 ± 0.12	6.12 ± 0.18	6.01 ± 0.10
Glucose (mEq/L)	134.45 ± 10.68	117.18 ± 23.90	92.82 ± 4.70	105.72 ± 8.23
HDL (mg/dl)	35.56 ± 2.76	41.58 ± 4.97	53.63 ± 5.28	53.52 ± 4.61
LDL (mg/dl)	20.68 ± 3.42	26.99 ± 2.52	19.32 ± 2.23	18.55 ± 1.58

**Table 8:** *In silico* toxicity prediction of compounds from the essential oil of *Thymus schimperi*.

No.	Compounds	Compound ID	In silico toxicity					
			h-ERG Blocker	HT	DILI	Ames toxicity	Cytotoxicity	MMP
1	Butanoic acid, 2-methyl-, methyl ester	13357	No	No	No	No	No	No
2	*α*-Thujene	17868	No	No	No	No	No	No
3	*α*-Pinene	6654	No	No	No	No	No	No
4	1,3,5-Cycloheptatriene, 7-ethyl-	561243	No	No	No	No	No	No
5	Camphene	6616	No	No	No	No	No	No
6	*β*-Pinene	440967	No	No	No	No	No	No
7	2,3-Diazabicyclo-[2.2.2] oct-2-ene	145130	No	No	No	No	No	No
8	3-Octanone	11527	No	No	No	No	No	No
9	Myrcene	31253	No	No	No	No	No	No
10	3-Octanol	246728	No	No	No	No	No	No
11	*α*-Phellandrene	443160	No	No	No	No	No	No
12	*γ*-3-Carene	26049	No	No	No	No	No	No
13	Cymene<ortho->	10703	No	No	No	No	No	No
14	D-Limonene	440917	No	No	No	No	No	No
15	Cineole<1, 8->	2758	No	No	No	No	No	No
16	Β-Ocimene<(Z)	5320250	No	No	No	No	No	No
17	*α*-Terpinene	7462	No	No	No	No	No	No
18	Terpinene<gamma->	7461	No	No	No	No	No	No
19	Dihydromyrcenol	29096	No	No	No	No	No	No
20	Terpinolene	11463	No	No	No	No	No	No
21	Linalool	6549	No	No	No	No	No	No
22	Sabina ketone<dehydro->	527426	No	No	No	No	No	No
23	*α*-Campholenal	249978459	No	No	No	No	No	No
24	Ocimene<allo->	5368821	No	No	No	No	No	No
25	Sabinol<*trans*-> (*trans* for OH vs. IPP)	564260	No	Yes	No	No	No	No
26	Isoborneol	64685	No	No	No	No	No	No
27	Methyl m-tolyl carbinol	110953	No	Yes	No	No	No	No
28	*α*-Terpineol	442501	No	No	No	No	No	No
29	Cyclohexane, 1-butenylidene-	556287	No	No	No	No	No	No
30	2-Isobutylideneamino-3-methylbutyronitrile	573025	No	Yes	No	No	No	No
31	Dodecane	8182	No	No	No	No	No	No
32	Dihydrocarvone<*trans*->	24473	No	No	No	No	No	No
33	3,5-Methanocyclopentapyrazole, 3,3a,4,5,6,6a-hexahydro-3a,4,4-trimethyl-	564375	No	No	No	No	No	No
34	1,4-Methano-1H-cyclopenta[d]pyridazine, 4,4a,5,7a-tetrahydro-8,8-dimethyl-, (1. alpha.,4. alpha.,4a. alpha.,7a. Alpha.)-	562380	No	No	No	No	No	No
35	Carvacrol, methyl ether	80790	No	No	No	No	No	No
36	Neral	643779	No	No	No	No	No	No
37	Limonen-10-ol	527143	No	Yes	No	No	No	No
38	Thymol	6989	No	No	No	No	No	Yes
39	Carvacrol	10364	No	No	No	No	No	No
40	Thymol acetate	241091509	No	No	Yes	No	No	No
41	Carvacrol acetate	80792	No	No	Yes	No	No	No
42	Sesquithujene<7-epi->	56927990	No	No	No	No	No	No
43	*β*-Bourbonene	324224	No	No	No	No	No	No
44	Caryophyllene (E-)	5281515	No	No	No	No	No	No
45	*β*-Gurjunene	6450812	No	No	No	No	No	No
46	*trans*-Alpha-bergamotene	6429302	No	No	No	No	No	No
47	Aromadendrene<allo->	91746537	No	No	No	No	No	No
48	3,5-Methanocyclopentapyrazole, 3,3a,4,5,6,6a-hexahydro-3a,4,4-trimethyl-	564375	No	No	No	No	No	No
49	Germacrene D	5317570	No	No	No	No	No	No
50	Amorphene<gamma->	12313019	No	No	No	No	No	No
51	Neryl isobutanoate	87203412	No	No	No	No	No	No
52	Viridiflorene	10910653	No	No	No	No	No	No
53	Geranyl isobutanoate	5365991	No	No	No	No	No	No
54	*β*-Sesquiphellandrene	519764	No	No	No	No	No	No
55	*β*-Vetivenene	14475467	No	No	No	No	No	No
56	*β*-Atlantol	181580	No	Yes	No	No	No	No
57	Tetracosane	12592	No	No	No	No	No	No

Toxicity profiles of compounds were analyzed using ADME web server (https://admet.scbdd.com) and vNN-ADMET web server. hERG: human ether-àgo-go-related gene, HT: hepatotoxicity, and DILI: drug-induced liver injury.

## Data Availability

All the data are included within the article.
